# Hearing and Speech Perception are Associated with A/T/N Biomarkers of Alzheimer's Disease

**DOI:** 10.1002/alz70856_107173

**Published:** 2026-01-07

**Authors:** Elodie Foster, Carolyn Herbert, Aaron Vosmeier, Savannah Hottle, Liana G. Apostolova, Jared R. Brosch, David G. Clark, Martin R Farlow, Sunu Mathew, Fredrick Unverzagt, Sophia Wang, Sujuan Gao, Charles W Yates, David Pisoni, Andrew J. Saykin, Shannon L Risacher

**Affiliations:** ^1^ Indiana Alzheimer's Disease Research Center, Indiana University School of Medicine, Indianapolis, IN, USA; ^2^ Department of Otolaryngology‐Head & Neck Surgery, Indiana University School of Medicine, Indianapolis, IN, USA; ^3^ Department of Neurology, Indiana University School of Medicine, Indianapolis, IN, USA; ^4^ Indiana Alzheimer's Disease Research Center, Indianapolis, IN, USA; ^5^ Center for Neuroimaging, Indiana University School of Medicine, Indianapolis, IN, USA; ^6^ Center for Neuroimaging, Department of Radiology and Imaging Sciences, Indiana University School of Medicine, Indianapolis, IN, USA

## Abstract

**Background:**

Hearing loss is linked to dementia, yet its relationship with Alzheimer's disease (AD) biomarkers remains unclear. This study examines associations between performance on auditory and speech perception measures with neuroimaging measures of neurodegeneration (N; cortical volume), amyloid (A), and tau (T) to aid early AD detection.

**Method:**

121 participants from the Indiana Alzheimer's Disease Research Center underwent auditory testing and neuroimaging. Auditory assessments included CUNY sentences, Speech‐in‐Noise (QuickSiN), and Letter Number Sequencing (LNS). Neuroimaging measures included MRI‐based cortical volume extracted with Freesurfer v6 (N), cortical Centiloid value from amyloid PET (A), and bilateral mean meta‐temporal tau standardized uptake value ratio from tau PET (T). A one‐way ANCOVA was used to compare differences in auditory tests between diagnostic groups, while partial Pearson correlations were used to assess associations of auditory tests with A/T/N biomarkers, covaried for age, sex, and total intracranial volume (*N* only).

**Result:**

The sample (age=71.90±6.6; 63.9% female) included 49 cognitively normal older adults, 40 with subjective cognitive decline, 23 with mild cognitive impairment (MCI) due to AD, 7 with MCI due to other conditions, and 2 with AD. Significant diagnostic differences were observed in SNR loss on QuickSiN (*p* = .006) and CUNY subtests, including AV words (*p* <.001), AV sentences (*p* = .037), and A words (*p* = .002).

Cortical amyloid correlated with SNR loss (r=.231, *p* = .045) and CUNY subscores, including auditory (Au) words (r=‐.272, *p* = .018) and word gain (r=‐.250, *p* = .031). LNS raw scores correlated with cortical volume (r=‐.311, *p* = .008). Associations were found between cortical volume and CUNY audiovisual (AV) words (r=.254, *p* = .023), A words (r=.395, *p* < .001), and sentence gain (r=‐.261, *p* = .020), but not with SNR loss. Meta‐temporal tau SUVR was associated with CUNY A words (r=‐.294, *p* = .021), A sent (r=‐.276, *p* = .031), word gain (r=.303, *p* = .016), and LNS raw scores (r=‐.276, *p* = .036), but not with SNR measures.

**Conclusion:**

Findings suggest that auditory and speech perception tests are associated with neuroimaging biomarkers of AD, supporting the potential utility of hearing assessments as early screening tools for AD‐related pathology. Further research with larger sample sizes is warranted to strengthen these findings and explore their clinical implications.

